# The Committee

**Published:** 1896-04

**Authors:** 


THE COMMITTEE appointed by the Texas State Medical Association at its last meeting to take steps to secure medical legislation, held a meeting in Dallas on the 25th of March, ult. We learn that at that meeting a draft of a bill to regulate the practice of medicine was presented by Dr. J. T. Wilson, of Sherman, chairman of the committee, and adopted, as expressing the views of the committee as to the needs of the occasion. Drs. T. D. Wooten, of Austin; J. F. Y. Paine, of Galveston; Bacon Saunders, of Fort Worth, and Knox, of Hillsboro, the other members, were present.

The bill, we learn, is framed very much after the form of the Ohio bill lately passed. It provides for representation on the board of examiners of all "schools" of medicine, so called. The regulars to have six, the homeopaths four and the eclectics two representatives each, or about in that proportion. The committee thought best, we learn, to not ask for a board of health at this time, hoping, if we can secure the passage of an act to require examinations and a license to practice, it will be an opening wedge, and we may be able to get further legislation later; that if we ask for too much at the hands of the legislature we are likely to get nothing.

This draft, we understand, is to be submitted to the State Medical Association at its meeting in Fort Worth on the 28th of this month, as part of the report of the committee. At the same time, it will be remembered, the East Texas Medical Society has, as we stated in our February number, adopted a bill gotten up by Dr. Waldert, or rather by a committee of the society, of which committee he is a member, and this bill, too, is to be submitted. From the two, perhaps, the best features will be taken, and the Association will finally decide upon the shape in which the bill will be presented to the legislature. The committee will, we learn, present it in person, and make a plea for its passage in the name of justice, humanity and common sense.

It looks as if the profession in Texas will yet have to take the soup, even if by enema. We don't want any homeopaths and eclectics, yet it looks like this: "No homeopaths, no regulate the practice." They have whipped us every time, but, according to our own way of thinking, there is a difference between acknowledging defeat in a manly, dignified manner, and accepting the consequences, and fawning to the power that defeated us, and joining forces with them to keep out all other quacks. In other words, if we can not get a bill to regulate the practice, without the *consent* of a large element of quacks, and their co-operation, let us do without it. It is forcing us to recognize homeopathy. We are by this like Huckleberry Finn was when his conscience smote him for not giving up the run away negro Jim. He reflected—"here am I harboring a negro that belongs to a good lady who never did me any harm; it is wrong, and if I don't give him up and do my duty I'll go to hell! that's what Miss Watson taught me in Sunday-school." After reflecting a long time, he finally said: "Well, if I've got to go to hell for not doing such a dirty cowardly thing as giving up poor Jim, why, I'll *go* to hell." Bully for Huck. And that's just the way we feel about getting a bill to regulate the practice with the *assistance* of, and by recognizing as equals—as physicians—a lot of the most blatant pretenders and frauds in the State (of course there are exceptions), we will let the practice go –where Huck said he'd go.

